# Intestinal Microbiota and Fecal Transplantation in Patients with Inflammatory Bowel Disease and *Clostridioides difficile*: An Updated Literature Review

**DOI:** 10.3390/jcm14155260

**Published:** 2025-07-25

**Authors:** Chloe Lahoud, Toni Habib, Daniel Kalta, Reem Dimachkie, Suzanne El Sayegh, Liliane Deeb

**Affiliations:** 1 Department of Internal Medicine, Staten Island University Hospital, Northwell Health, 475 Seaview Avenue, Staten Island, New York, NY 10305, USA; 2 Division of Gastroenterology and Hepatology, Staten Island University Hospital, Northwell Health, 475 Seaview Avenue, Staten Island, New York, NY 10305, USA

**Keywords:** fecal transplant, donor feces infusion, *Clostridioides difficile*, inflammatory bowel diseases, ulcerative colitis, Crohn’s disease

## Abstract

**Background/Objectives**: Inflammatory bowel disease (IBD) is characterized by chronic relapsing and remitting inflammation of the gastrointestinal tract. Fecal microbiota transplantation (FMT) has emerged as an FDA-approved treatment for recurrent *Clostridioides difficile* infections (CDIs), with promising potential in patients with IBD. This manuscript aimed to provide a comprehensive and updated review of the available literature on fecal microbiota transplantation, its clinical use in IBD in general, as well as in patients with IBD and CDI. **Methods**: An extensive literature search was performed from October 2024 to March 2025. All publications available within PubMed, Medline, Embase, Google Scholar, and Cochrane databases were reviewed. All original articles, case reports, review articles, systematic reviews, and meta-analyses were included. Qualitative and quantitative data were both extracted. **Discussion**: Intestinal microbiota is an integral part of the human body, and dysbiosis (an imbalance in the gut’s microbial community) has been linked with several pathologies. Dysbiosis in IBD is marked by reduced beneficial bacteria and increased pro-inflammatory pathogens, contributing to mucosal damage and immune dysregulation. FMT has emerged as a solution to dysbiosis, with the first case recorded in 1917. FMT has been successful in treating patients with CDI. The diagnostic value of the gut microbiome is currently being explored as a possible therapeutic approach to IBD. Several studies have assessed FMT in patients with IBD and CDI with promising results in both ulcerative colitis (UC) and Crohn’s disease (CD) but varying efficacy based on administration routes, donor selection, and processing methods. In the context of recurrent CDI in patients with IBD, FMT demonstrates a high cure rate and potential benefit in concurrently improving IBD activity. However, risks such as IBD flare-ups post-FMT remain a concern. **Conclusions**: FMT holds promising potential in the management of CDI in patients with IBD. By restoring microbial diversity and correcting dysbiosis, FMT offers a novel, microbiota-targeted alternative to conventional therapies. While data support its efficacy in improving disease remission, variability in outcomes underscores the need for standardized protocols and additional large-scale, controlled studies. Continued research efforts into donor selection, treatment regimens, and long-term safety will be critical to optimizing FMT’s role in IBD and CDI care as well as improving patient outcomes.

## 1. Introduction

Inflammatory bowel disease (IBD) is characterized by chronic relapsing and remitting inflammation of the gastrointestinal (GI) tract and encompasses two main entities differentiated by their location and depth of intramural bowel involvement: Crohn’s disease (CD) and ulcerative colitis (UC) [1]. The gut microbiota, referring to the entire population of microorganisms that colonize the GI tract, bears a significant functional role in maintaining nutrient metabolism, intestinal barrier function, and prevention of colonization of pathogenic microorganisms. Traditional therapies for IBD aim to suppress inflammation and induce or maintain remission. However, these treatments often come with significant side effects, variable efficacy, and sometimes limited impact. There has been growing interest in microbiota-targeted therapies as a novel approach to managing IBD, with the potential to address not only symptoms but also one of the disease’s key contributing factors. Fecal microbiota transplantation (FMT) has emerged as a solution to dysbiosis, an imbalance in the gut’s microbial community [2]. FMT is an FDA-approved treatment for recurrent CDI, with promising therapy potential for *C. difficile* in patients with IBD. Patients with IBD and CDI are at higher risk for prolonged hospitalizations and severe disease flares, underscoring the importance of effective therapeutic strategies for this subset of patients.

In this manuscript, we aim to provide a comprehensive and updated review of the available literature on fecal microbiota transplantation, its clinical use in IBD in general, as well as in patients with IBD and CDI. By examining current evidence, we highlight both the therapeutic potential and the gaps that remain in integrating FMT into routine clinical practice for these patients.

## 2. Materials and Methods

An extensive literature search was performed from October 2024 to March 2025. All publications available within PubMed, Medline, Embase, Google Scholar, and Cochrane databases were reviewed for published and relevant literature. All original articles, case reports, review articles, systematic reviews, and meta-analyses were included.

The following search terms were employed: “intestinal microbiota”, “gut microbiome”, “fecal microbiota”, “fecal microbiome transplant”, “fecal transfusion”, “donor feces infusion”, “stool transplant”, “fecal bacteriotherapy”, “*Clostridioides difficile*”, “*Clostridioides difficile* infection”, “ulcerative colitis”, “Crohn’s disease”, “inflammatory bowel disease”, “IBD”, “Vowst”. Qualitative and quantitative data were both extracted. Studies reviewed were limited to those written in English. To note, the available literature lacks uniformity on practical application of FMT such as patient and donor selection, dose, route, frequency, and long-term follow-up policy. We discuss mechanisms of FMT and information gaps throughout this review.

## 3. Discussion

### 3.1. Role of Intestinal Microbiota in Health and Immune Development

Intestinal microbiota is an integral part of the human body. On average, there are around 300–500 bacterial species that inhabit the intestines, which contain nearly 2 million genes [3]. Collectively there is around 10 times the number of bacteria than human cells in a person [4]. It can harvest inaccessible nutrients from the body, changing body composition as well as synthesis of vitamins otherwise not produced by the body itself. It can result in higher body mass indexes in those with certain gut microbiota [5]. It can also impact drug metabolism, impacting the bioavailability of active medications. A novel study performed on mice revealed that oxalate metabolism, for example, can be impacted by the gut microbiota leading to an increased risk for nephrolithiasis [6]. There are studies that reveal that the gut microbiota can influence the development of the innate and adaptive immune system, with some bacteria having the ability to influence the expression of antimicrobial compounds released by the innate immune system, thereby indirectly influencing the human hosts’ susceptibility to colonization from other pathogens [7,8]. Some bacteria provide specific resistances, such as segmented filamentous bacterium, which were found to induce Th17 cells in the lamina propria. This results in enhanced resistance to *Citrobacter rodentium* by regulating inflammation and anti-microbial defenses [9].

At birth, the intestinal tract is sterile and undergoes initial microbial colonization through maternal and environmental exposure. Subsequent maturation of the gut microbiome is influenced by feeding practices and continued environmental interactions [10]. Development of the intestinal microbiota starts early to ensure the creation of a proper immune system. This is commonly known as the hygiene hypothesis, which stems from the idea that the recent increase in atopic disorders could be due to the decreased number of infections in early childhood. Many studies have shown that animals not exposed to germs generally have an under-developed immune system. This can be characterized by a decrease in the size and amount of the Peyer’s patches mesenteric lymph nodes and lymphoid particles [11]. Factors that are known to impact the colonization of the bowel are mode of delivery (C-section compared to vaginal delivery), gestational age, level of sanitation, and diet (breast milk vs. formula) [12]. The bacteria in the gut do adapt and change over time, which allows for the body to strengthen its intestinal immune system. There are studies that show, however, that without early exposure, mice might miss an essential period of immune and mucosal development. This was especially noted in transcriptional profiles in both the jejunum and colon of mice that were germ-free compared to conventionally raised mice [13]. It has even been found that maternal exposure to microbiota could also impact immune system development. This could even be seen in mice that were otherwise germ-free, meaning that just a transient exposure could bolster the immune system and minimize inflammation [14]. These effects are not limited to infections, extending to lifetime diseases. One study showed that exposure to microbiota in the setting of gastroenteritis or respiratory infections have been found to be protective factors for inflammatory bowel disease [15].

### 3.2. Fecal Microbiota Transplantation

Considering the plethora of studies citing the importance of intestinal microbiota, scientists have been focusing on solutions to dysbiosis. General first-line agents have been probiotics and prebiotic foods; however, they can be limited in their efficacy. FMT has become increasingly popular as a solution. Records of fecal transplantation can date back to China in the 300s AD where they would use it to treat severe cases of diarrhea and food poisoning [16]. This was also investigated in the Middle East with the Bedouin people who consumed camel stool for dysentery treatment, as well as in Italy with farm animals, where they would transplant intestinal content from healthy animals to sick ones [17,18,19]. The first modern case of fecal transplant was in 1917, during the First World War. The scientist Dr. Nissle isolated an *E. coli* strain which was eventually named after him, *Escherichia coli* strain Nissle 1917 or EcN, from a German soldier’s stool. Dr. Nissle noticed this soldier was one of the few that did not contract shigella, which was running rampant in the region where they were deployed in Germany [18]. EcN was eventually found to not only be protective against other gastroenteritis infections, but also against chronic inflammatory conditions such as inflammatory bowel diseases [20].

Early trials in FMT were met with resistance, with bacteriologist Stanley Falkow being fired in the 1950s for trying to research its effects on surgical patients post-antibiotics. Preliminary data from his studies showed radical success. Future studies performed were also very successful. There was a study performed a year later which was successfully able to treat 94% of patients with pseudomembranous colitis with FMT, all of which had poor prognoses at the time [17].

Eventually, it was found that FMT was especially helpful for CDI. Multiple randomized controlled trials showed that FMT cured at least 90% of patients with CDIs [17]. One study showed FMT worked for patients with recurrent CDIs and who failed a full course of antibiotics. FMTs have also been found to help reverse metabolic syndrome in rats. The study was profound in that previous research which had similar findings required surgical extraction of cecal bacteria; however, it was found that simple fecal content was sufficient [21]. Both also indicate that intestinal microbiota has a major impact in metabolism. As time has progressed, there have been numerous case reports that show remission of many diseases post-FMT. These include Parkinson’s disease, multiple sclerosis, and idiopathic thrombocytopenic purpura [17].

Moreover, the beneficial effects of FMT extend to other organ systems, such as the cardiovascular system. In a study performed on mice fed a Western diet to promote atherosclerosis, a subgroup was given 8 weeks of *A. muciniphila*, and it was found that the atherosclerotic lesions were reversed. This was achieved by preventing endotoxins from being released into the body, which would have caused inflammation by inducing tight junction proteins, and thereby decreases gut permeability [22].

### 3.3. Microbiome-Based Diagnostics in IBD

The diagnostic value of the gut microbiome in IBD is currently being explored. Patients with IBD exhibit higher levels of mucosa-adherent bacteria, particularly in inflamed areas such as the terminal ileum and colorectum, and disease activity correlates with bacterial number and abundance [23]. Sampling these areas can be helpful in identifying the predominant pathogen, which can assist with diagnosis, prognosis, treatment response prediction, and disease activity monitoring for IBD [24]. Although histopathology remains the gold standard for IBD diagnosis and monitoring, providing detailed mucosal information, colonoscopy to obtain biopsies is still invasive and can cause discomfort and bleeding, along with possibly introducing sampling bias due to limited mucosal sampling [25]. Mucosal brush samples are less invasive, covering a wider area and yielding more bacterial DNA with less host DNA contamination [26,27]. Laser capture microdissection (LCM) can also be used and provides high-quality RNA for analysis, especially if performed under argon and using ethanol to preserve RNA integrity [28].

Fecal samples remain the only non-invasive method but may not fully represent the entire gut microbiota due to regional variations in microbial composition [29]. Saliva offers a potential non-invasive alternative, with mouthwash collection being a convenient option [30,31,32,33]. Other fluids like urine and serum have also been used [34,35].

Fecal samples from patients with IBD reveal altered microbial diversity, with decreased Bacteroides and Firmicutes and increased Actinobacteria and Proteus [36]. Mucosal tissue and stool samples show increased *Pasteurella*, *Veronococcus*, *Neisseria*, *Clostridium* spp., and *Escherichia coli* and decreased *Pseudomonas*, *Clostridia*, *E. faecalis*, *Rhodococcus*, *Brault’s* spp., *Rumenococcus*, and *Trichophyton* spp. in patients with CD [37]. *Faecalibacterium prausnitzii* and *Rothbyrrhizobium* are reduced in CD, potentially increasing pro-inflammatory cytokines and intestinal inflammation [38]. Postoperative endoscopic recurrence of ileal CD is associated with changes in microbial composition, including increased *Proteobacteria*, *Lachnospiraceae*, *Ruminococcus*, *Fusobacteria* [39], and adherent-invasive *Escherichia coli* (AIEC) and decreased *Faecalibacterium* [40,41]. These microbes may serve as potential therapeutic targets for preventing recurrence. Table 1 summarizes these findings.

Viruses also play a role in the pathogenesis of IBD. For example, Caudovirales bacteriophages are associated with increased interferon-γ (IFN-γ) production, exacerbating especially ulcerative colitis [42,43]. The hepatitis B virus X protein (HBx), encoded by the Orthohepadnavirus genus, can disrupt the intestinal barrier [44]. Murine norovirus (MNV) infection also decreases tight junction molecule expression and increases epithelial cell death [45].

Additionally, intestinal fungi influence the microbiota and immune responses. Yeasts can cause abnormal CD4+ T cell reactivity in CD [46,47,48]. *Debaryomyces hansenii* impairs mucosal repair [49], and *Malassezia restricta* exacerbates colitis [50]. *Candida albicans* increases pro-inflammatory cytokine production in IBD [51]. Microsporidia can increase epithelial permeability, reduce wound repair, and disrupt tight junction proteins, potentially contributing to IBD pathogenesis [52,53].

Microbial species identification holds promise for discovering biomarkers for CD, UC, or both. For instance, *Akkermansia muciniphila* has been proposed as a potential diagnostic biomarker for pediatric CD [54]. *E. coli* and *F. prausnitzii* are useful for IBD phenotypic categorization [55]. Whole-metagenome sequencing of patients with CD has highlighted single-nucleotide variants (SNVs) in Bacteroides species (*B. ovatus*, *B. vulgatus*, and *B. uniformis*) as potentially significant diagnostic markers [56]. Reduced *Firmicutes* (*Clostridiales*) levels correlate with IBD severity, and when combined with calprotectin and CD activity index, *Clostridiales* can predict treatment success [57]. *Enterococcus* and *Lactobacillus* show increased abundance in UC, suggesting their potential as biomarkers [58]. *Clostridium cluster XVIII*, *Thomasclavelia ramosum*, *T. spiroforme*, and *T. saccharogumia*, identified via two-dimensional polymerase chain reaction (2D-PCR), may aid in early IBD diagnosis and monitoring [59]. Table 2 summarizes these findings.

Baseline abundances of *Bifidobacterium*, *Clostridium colinum*, *Eubacterium rectale*, uncultured *Clostridiales*, and *Vibrio*, coupled with lower *Streptococcus mitis* levels, predict positive response to anti-TNF-α treatment, as indicated by reduced calprotectin [60]. Post-FMT responders with UC show increased alpha diversity, *Clostridiales*, and *Bacteroides* [61]. Higher *Bifidobacterium* levels, potentially treatment-related, contrast with lower levels in irritable bowel syndrome (IBS) [62]. Sixteen functional pathways and 12 microbial species, including *Roseburia hominis* and *Dialister invisus*, are associated with future treatment intensification, suggesting their potential for personalized IBD management [63].

Despite this potential, several challenges remain. Interindividual variation, technological limitations, and the dynamic nature of the gut microbiome necessitate longitudinal studies [64,65]. Furthermore, the gut microbiome’s role in colorectal cancer (CRC), an IBD complication, suggests its potential as an early detection biomarker [66]. Receiver operating characteristics (ROC) analysis, machine learning, multi-omics approaches, and random forest (RF) models are valuable tools for biomarker discovery and treatment outcome prediction [27,58,64,67,68,69].

### 3.4. Clinical Use of FMT in IBD

#### 3.4.1. Pathogenesis of the Gut Microbiome in IBD

The gut microbiota significantly impacts overall health and is directly linked to gastrointestinal diseases such as IBD. FMT is an FDA-approved treatment for recurrent *Clostridioides difficile* infections and has gained attention for treating irritable bowel syndrome and IBD [70]. Although the exact role of the gut microbiome in IBD pathogenesis is unclear, dysbiosis—an imbalance in the gut microbial community—is considered a key initiating factor [71]. This dysbiosis is influenced by genetic, environmental, and immune factors [71]. It is characterized by reduced levels of beneficial short-chain fatty acid (SCFA)-producing bacteria and increased harmful metabolites from intestinal pathogens. This imbalance leads to elevated pro-inflammatory mediators, mucosal damage, increased permeability, disrupted immune homeostasis, and impaired immune regulation, potentially contributing to autoimmunity [58].

#### 3.4.2. Therapeutic Role of Fecal Microbiota Transplant in IBD

Redirecting the gut microbiota has been investigated as a possible therapeutic approach to IBD. As current treatment causes immunosuppression and predisposes patients to infections, FMT seems a better alternative with fewer side effects. FMT mainly involves transferring fecal material from a healthy donor to a patient with IBD [24,72]. It is thought to be superior to other therapies targeting gut microbiota, such as prebiotics, probiotics, or antibiotics, as it offers a complete functional ecosystem from a healthy donor.

Current FMT administration routes involve the upper digestive tract (nasogastric, nasoduodenal, and nasojejunal tubes), the lower digestive tract (enema and colonoscopy), and oral capsules [73]. Studies show promising results in UC, with increased remission rates with FMT enemas and capsulized FMT compared to controls [74,75]. A single fresh FMT was also found to induce long-term remission in active UC, increasing *Bacteroidetes* and decreasing *Proteobacteria* [76]. An analysis of four studies (277 total participants) demonstrated FMT’s efficacy in UC, with a 37% remission rate in FMT recipients at week eight compared to 18% in controls, effectively doubling the remission rate [73]. However, due to UC’s heterogeneity, FMT response varies [77]. Patients achieving remission post-FMT exhibited greater microbial diversity in mucosal and fecal samples, along with increased SCFA biosynthesis [78]. Other studies recognized FMT’s efficacy in mild-to-moderate UC remission [79] but acknowledged the need for further research regarding safety, administration methods, bacterial dose, frequency, and long-term prognosis.

Data on FMT for Crohn’s disease are somewhat more limited than UC [80]. Published literature has mixed results; some studies report remission while others report no effect. Dina et al. [81] report the case of a 26-year-old man with Crohn’s disease, who presented for colitis with recurrent peri-anal fistulas and bloody diarrhea, underwent four FMT infusions (at 0, 4, 6, and 8 weeks), and remained in clinical and endoscopic remission at repeat colonoscopy. Similarly, Forgon et al. [82] report the case of a 35-year-old man who presented with recurrent Crohn’s colitis, received FMT, and remained in remission for 6 months afterwards. On the other hand, in a pilot study by Vermeire et al. [83] in 2012, four patients with CD refractory to treatment with corticosteroids, immunomodulators, and anti-TNF antibodies underwent FMT. Eight weeks after FMT, no clinical or endoscopic efficacy was observed. FMT also shows promising results in refractory CD, with one study reporting initial clinical improvement and remission rates of 86.7% and 76.7%, respectively [84]. Other studies have shown FMT’s efficacy in resolving severe complications in refractory CD and improving species richness, leading to a 66.7% remission rate [81,85,86]. Some FMT recipients showed limited donor–recipient flora similarity, suggesting a single FMT may be insufficient for substantial change, though FMT still outperformed controls in endoscopic severity and C-reactive protein (CRP) levels [87]. While promising results have been observed in severe IBD [88], efficacy remains variable. Overall, many more research efforts are needed to assess efficacy in this subpopulation.

Current studies aim to assess whether it is better to use single versus multi-donor FMT and fresh versus frozen FMT. While a single FMT may not drastically alter the gut microbiome in all patients with CD, it can still be beneficial [87]. Multi-donor FMT strategies aim to enhance microbial diversity and minimize individual donor variability [53]. While fresh FMT is often used, ongoing research compares its efficacy to frozen FMT, considering factors like anaerobic bacteria survival [27,29]. The FOCUS study utilized a multi-donor FMT approach for UC, aiming to improve microbial diversity and minimize risks associated with single-donor FMT. Results showed that multi-donor FMT recipients achieved and sustained similar alpha-diversity levels compared to single-donor recipients [53]. While fresh and frozen FMT show comparable efficacy and safety in *C. difficile* infection (CDI), there are limited comparative data in IBD [26]. Given the potential loss of beneficial obligatory anaerobes like *Faecalibacterium prausnitzii* during aerobic FMT processing, anaerobic FMT production has been suggested for improved UC outcomes [27]. However, controlled studies comparing anaerobic and traditional FMT processing are needed to definitively assess efficacy and account for confounding donor and patient factors.

Combining probiotics with FMT may enhance treatment for colitis, as demonstrated by increased *Firmicutes* and decreased *Bacteroidetes* in mice treated with *L. plantarum* and FMT [89]. FMT in combination with tislelizumab (an anti-programmed death receptor 1 (PD-1) monoclonal immunoglobulin G-4 antibody [90]) and fruquintinib (a novel potent anti-vascular endothelial growth factor (VEGF) tyrosine kinase inhibitor [91]) has also shown promise for refractory metastatic colorectal cancer [92]. Washing microbiota transplantation (WMT), a novel FMT technique, has shown efficacy in treating CD and improving glycemic variability, and may be more cost-effective than infliximab in the long term [93,94,95]. Fecal virome transplantation (FVT) is also being explored, with some success in treating CDI and metabolic disorders, but with potential for exacerbating IBD in some cases [96,97,98,99]. Despite its potential, FMT has limitations, including temporary effects, pathogen transmission risk, and logistical challenges related to storage and donor selection [70,100,101].

### 3.5. FMT in IBD Patients with Recurrent Clostridioides difficile Infection

*Clostridioides difficile* is a gram-positive, spore-forming bacterium, which causes antibiotic-associated colitis [102]. FMT has been successful in treating infection with *C. difficile* for over 60 years [103]. FMT is unique as it increases the diversity of fecal bacterial populations in the recipients and leads to long-term engraftment in recipients with *C. difficile* infection, unlike probiotics [72,104,105]. It is a potent and promising therapy for *C. difficile* in IBD.

The first report of FMT in UC goes back to 1989: Justin Bennet, a physician suffering from severely active UC self-administered a fecal load from a healthy donor by an enema and achieved a 6-month symptom-free remission afterwards [106].

Since then, FMT has become an approved treatment for recurrent and refractory *C. difficile* infection [107]. However, its use in patients with UC still has significant gaps in the understanding of the exact mechanisms of action. Porcari et al. [108] completed a retrospective cohort study in 2023 in which they evaluated 35 patients with UC who received FMT for recurrent CDI, and the primary outcome was a negative *C. difficile* toxin 8 weeks after FMT. In this study, 91% of patients were cured (16 patients after single FMT and 13 patients after repeat FMT). Repeat FMT was significantly associated with sustained cure of CDI compared with single FMT (84% compared to 50%, *p*-value = 0.018). There were no serious adverse events reported after FMT [108]. In addition, 69% of patients experienced remission or improvement of UC activity after they received FMT.

In 2018, Mintz et al. [109] studied colonoscopic FMT in an open-label prospective study among three patients with more than two recurrences of CDI (11 patients) and/or patients with UC not responsive to therapy (eight patients). They divided the patients into three groups: CDI-only, CDI + UC, and UC-only patients. They performed microbiome analysis and ribosomal RNA gene sequencing pre-, 1 week post-, and 3 months post-FMT. CDI-only and CDI + UC groups had marked decreases in the strictly anaerobic Bacteroidetes phylum and two Firmicutes sub-phyla, as well as increases in the microaerophilic Proteobacteria phylum and the Firmicutes/Bacilli group. It was found that the microbial genomes in CDI + UC recipients resembled more closely those in the CDI-only recipients than the UC-only recipients.

Several studies have assessed FMT in patients with IBD and CDI, with promising results. In an open-label prospective multicenter cohort study performed by Allegretti et al. [110] in 2020, 50 patients with IBD (15 with CD and 35 with UC) who had two or more CDI episodes within the last 12 months were enrolled. They all received a single FMT from a healthy universal donor, by colonoscopy. At 6 months, 73% of patients with CD and 62% of patients with UC had IBD improvement [110]. In a retrospective study performed by Meighani et al. [111] between 2012 and 2014, 201 patients with recurrent CDI underwent FMT (20 of them had concurrent IBD). The overall response rate among the patients with IBD and CDI was 75% at the 12-week follow-up (*p*-value = 0.13). In addition, Tariq et al. [112] reported an overall cure rate of CDI after FMT to be 80% among patients with IBD (54 patients with CD and 89 patients with UC) and recurrent CDI. Azimirad et al. [113] have also found comparable rates; they enrolled seven patients with UC and one patient with CD who received at least one FMT for treatment of recurrent CDI in 2020, with an overall cure rate of 75%. Comparably, Ianiro et al. [114] completed a single-center prospective cohort study on 18 patients with IBD and recurrent CDI receiving FMT between 2016 and 2020. An amount of 94% of the patients had negative *C. difficile* toxin and clinical improvement at 8 weeks.

Similarly, Khoruts et al. [115] completed a retrospective chart review of 272 patients who had recurrent CDI and did not clear the infection after one extended antibiotic course. Among these patients, 74.4% of those with IBD cleared CDI after FMT, compared to 91.1% of those without IBD (*p*-value = 0.0018) [115]. To note, 25.6% of patients with IBD had a clinically significant IBD flare after FMT [115]. Table 3 summarizes these findings.

Multiple case reports are available in the medical literature. Aratari et al. [116] describe the case of a 28-year-old man with chronic refractory UC who received FMT and infliximab infusions afterwards and successfully achieved and sustained remission. Another case of an 82-year-old female by Nanki et al. sheds light on the efficacy of FMT in restoring intestinal microbiota and curing CDI in UC [102].

This has also been studied in the pediatric population by Cho et al. [117], who performed a retrospective chart review of eight pediatric patients with IBD (six with UC, one with CD, and one with unspecified IBD) and recurrent CDI and received FMT. The cure rate observed in this study was 75% at 60 days.

### 3.6. FMT Adverse Events

While upper gastrointestinal (GI) administration of FMT has a higher overall incidence of adverse events, lower GI delivery carries a greater risk of severe events. Common, moderate side effects include abdominal cramping, mild diarrhea, bloating, GI discomfort, vomiting, and fever, which are typically self-limiting. Other serious adverse events include infection, GI inflammation (appendicitis, diverticulitis), and procedural complications like aspiration (upper GI) and bowel perforation (endoscopy) [28]. Allergic reactions to donor antigens are a theoretical concern in patients with a history of severe allergies or anaphylaxis [101]. The long-term effects of gut microbiota manipulation are still unclear, necessitating careful patient counseling and continued monitoring.

Some studies in the literature describe IBD flares following FMT. De Leon et al. reported the case of a 78-year-old male patient with quiescent ulcerative colitis (not requiring mesalamine or immunosuppressive medications for nearly 20 years) who was referred for FMT after three episodes of CDI. He received FMT from his wife as a donor through a colonoscopy. Nine days after FMT, the patient had an acute UC flare with abdominal cramping, bloody mucoid stools, and tenesmus, with friability from the rectum to the splenic flexure on sigmoidoscopy (new findings since colonoscopy at the time of FMT) [88]. This case emphasizes the need for prospective, controlled trials of FMT in IBD patients infected with CDI to evaluate safety, efficacy, and to delineate risks and benefits.

A systematic review performed by Qazi et al. [118] in 2017 included 29 studies and 514 FMT-treated patients with IBD. The pooled rate of worsening IBD after FMT was 14.9%. Interestingly, Qazi et al. [118] report a higher worsening IBD rate when FMT was performed for CDI (22.7%) compared to when it was performed for IBD (11.1%). Also, the rate of worsening IBD was higher after lower-GI FMT delivery (16.5%) compared with upper-GI delivery (5.6%).

Further research is needed to optimize FMT protocols, including administration route, frequency, donor selection, and long-term effects [119]. Understanding the complex interplay between gut microbes, intestinal immunity, and IBD remains crucial for developing personalized and effective treatments [73,120,121]. Emerging technologies like multi-omics approaches and machine learning are aiding in biomarker discovery and treatment outcome prediction [53].

### 3.7. Clinical Use of Vowst in Recurrent CDI

More standardized microbiome therapies have been emerging and have been shown to be efficacious and safe in recurrent CDI. In April 2023, the US Food and Drug Administration (FDA) approved Vowst for the prevention of recurrence of CDI in individuals 18 years of age and older, following antibacterial treatment for recurrent CDI [122]. Vowst is the first live biotherapeutic and fecal microbiota product that is taken orally. Vowst contains live bacteria, such as *Firmicutes* spores, and is made from human fecal matter [123]. The Vowst regimen dose is four capsules orally once a day for three consecutive days [122].

The ECOSPOR III randomized, double-blind, placebo-controlled clinical trial performed in 2023 evaluated the safety of Vowst [124]. The trial included 182 adult patients in the US and Canada who had three or more recurrent CDI episodes, with the primary outcomes being CDI recurrence rate at 8 weeks after treatment. The trial had two main groups: the Vowst group (n = 89) and placebo group (n = 93). Eight weeks after treatment, CDI recurrence in the Vowst group was lower compared to the placebo group (12.4% compared to 39.8%, *p*-value < 0.001) [124]. Across all patients, the Vowst group had a lower relative risk of recurrence when compared with the placebo group (RR = 0.32, *p*-value < 0.001) [124]. In this trial, Vowst was generally well tolerated, and no serious adverse events were reported [124]. Vowst’s efficacy was sustained at the 24-week post-treatment follow-up with a lower relative risk in the Vowst group (RR = 0.46, *p*-value < 0.001) [125]. This constitutes a significant step forward in advancing patient care and outcomes, as it offers a patient-friendly, orally administered, non-invasive, efficacious alternative to patients.

Research is very scarce as this is an emerging therapy. Among the potential risks, the donated stools are tested for transmissible pathogens; however, there may be risk of transmitting infectious agents as well as possible food allergens [122]. Some of the reported side effects by Vowst recipients are abdominal bloating, constipation, diarrhea, fatigue, and chills [122]. Further targeted research efforts are needed to evaluate Vowst’s efficacy in specific patient populations, especially in patients with IBD, its cost-effectiveness, and its integration into routine clinical practice.

## 4. Limitations

Although minimal side effects are expected in FMT for *C. difficile* infection, some published cases in the literature have reported adverse events.

In a case series performed by Fischer et al. [126], the authors report that 2.9% of patients with IBD treated with FMT for CDI were hospitalized for IBD flare after FMT. De Leon et al. [88] report a case of a transient flare of UC after FMT for recurrent CDI in a patient who had been quiescent for more than 20 years. However, FMT for patients with IBD without CDI has only had mild and self-limiting side effects. In a systematic review performed by Colman et al., no serious adverse events were reported in any of the studies included, where all adverse events were mild to moderate such as fever and abdominal tenderness and were self-limited. These findings suggest that the potential for side effects in FMT may be greater in IBD with recurrent CDI and shed light on the need for caution in treating CDI with FMT in patients with IBD.

IBD is clearly associated with dysbiosis, and the available data suggest there may be a role for manipulating the intestinal microbiota in the treatment of these devastating diseases [80]. It is also important to note that all published literature about FMT varies on the method of delivery, method of stool filtration, frequency of administration, bacterial dose, and most do not control for donor factors such as diet [80].

## 5. Conclusions and Future Directions

In conclusion, while FMT remains a generally safe and effective therapy for recurrent CDI in the general population, it holds promising potential in the management of CDI in patients with IBD. Current evidence demonstrates high efficacy for FMT in resolving recurrent CDI in both ulcerative colitis and Crohn’s disease patients, with some reports suggesting concurrent improvement in IBD disease activity. By restoring microbial diversity and correcting dysbiosis, FMT offers a novel, microbiota-targeted alternative to conventional therapies. While data support its efficacy in improving disease remission, variability in outcomes underscores the need for standardized protocols and additional large-scale, controlled studies. Continued research efforts into donor selection, treatment regimens, and long-term safety will be critical to optimizing FMT’s role in IBD and CDI care as well as improving patient outcomes.

## Figures and Tables

**Table 1:** Microbial changes observed in IBD including specific alterations in microbial diversity, changes in mucosal tissue, and stool samples.

Sample Type	Microbial Changes in Patients with IBD	Microbial Changes in Patients with CD	References
Fecal samples	Decreased Bacteroides and Firmicutes; increased Actinobacteria and Proteus	N/A	[36]
Mucosal tissue and stool samples	N/A	Increased *Pasteurella*, *Veronococcus*, *Neisseria*, *Clostridium species*, *Escherichia coli*; decreased *Pseudomonas*, *Clostridia*, *Enterococcus faecalis*, *Rhodococcus*, *Brault’s* species, *Ruminococcus*, *Trichophyton* species	[37]
Specific bacteria	N/A	Reduced *Faecalibacterium prausnitzii* and *Rothbyrrhizobium*, potentially increasing pro-inflammatory cytokines and intestinal inflammation	[38]
Postoperative recurrence of ileal CD	N/A	Increased *Proteobacteria*, *Lachnospiraceae*, *Ruminococcus*, *Fusobacteria*, adherent-invasive *Escherichia coli (AIEC)*; decreased *Faecalibacterium*	[39,40,41]

**Table 2:** Potential diagnostic biomarkers for CD and UC based on microbial species identification.

Diagnostic Biomarker	Relevance in IBD	References
*Akkermansia muciniphila*	Biomarker in pediatric CD	[54]
*Escherichia coli* and *F. prausnitzii*	IBD phenotypic categorization	[55]
*Bacteroides* species (SNVs)	Diagnostic markers	[56]
*Firmicutes* (*Clostridiales*)	Reduced levels correlated with IBD severity	[57]
*Enterococcus*	Increased in UC	[58]
*Lactobacillus*	Increased in UC	[58]
*Clostridium cluster XVIII*, *Thomasclavelia ramosum*, *T. spiroforme*, and *T. saccharogumia*	Aid in early IBD diagnosis and monitoring	[59]

**Table 3:** Studies assessing the efficacy of FMT in patients with IBD and CDI.

Study	Study Design	Patient Population	FMT Administration	Reported Outcomes	References
Allegretti et al. (2020)	Open-label prospective multicenter cohort	50 patients with IBD (15 CD, 35 UC) with ≥2 CDI episodes in 12 months	Single FMT by colonoscopy from a healthy universal donor	73% patients with CD and 62% patients with UC had IBD improvement at 6 months	[110]
Meighani et al. (2012–2014)	Retrospective study	201 patients with recurrent CDI (20 of them had concurrent IBD)	Not specified	75% response rate at the 12-week follow-up	[111]
Tariq et al. (2023)	Systematic review and meta-analysis	457 adult IBD patients with CDI	Single and multiple FMTs	78% CDI resolution after first FMT; 88% overall cure rate with single and multiple FMTs	[112]
Azimirad et al. (2020)	Prospective study	7 patients with UC and 1 patient with CD	At least one FMT for treatment of recurrent CDI	75% cure rate	[113]
Ianiro et al. (2021)	Single-center prospective cohort study	18 patients with IBD, recurrent CDI	At least two fecal infusions	94% of the patients had negative *C. difficile* toxin and clinical improvement at 8 weeks	[114]
Khoruts et al. (2016)	Retrospective chart review	272 patients who had recurrent CDI	Single FMT	74.4% of those with IBD cleared CDI after FMT, compared to 91.1% of those without IBD	[115]

## Data Availability

Not applicable.

## Abbreviations

The following abbreviations are used in this manuscript:

IBD	inflammatory bowel disease
CDIs	*Clostridioides difficile* infections
FMT	fecal microbiota transplantation
CD	Crohn’s disease
UC	ulcerative colitis
SCFA	short-chain fatty acid
LCM	laser capture microdissection
DNA	deoxyribonucleic acid
RNA	ribonucleic acid
AIEC	adherent-invasive *Escherichia coli*
IFN-γ	interferon-γ
SNV	single-nucleotide variants
MNV	murine norovirus
